# Supplementary material for: Relative Validity and Reproducibility of a Web-Based Semi-Quantitative Food Frequency Questionnaire in the Danish Diet, Cancer, and Health—Next Generations MAX Study
Source: Nutrients. 2023 May 19;15(10):2389. doi: 10.3390/nu15102389 (PMC10222214; doi:10.3390/nu15102389)
Supplement: Supplementary file 1 [file nutrients-15-02389-s001.zip › FFQ12months for energy-adjusted fat, protein, carbohydrates, iron, and vitamin C.pdf]

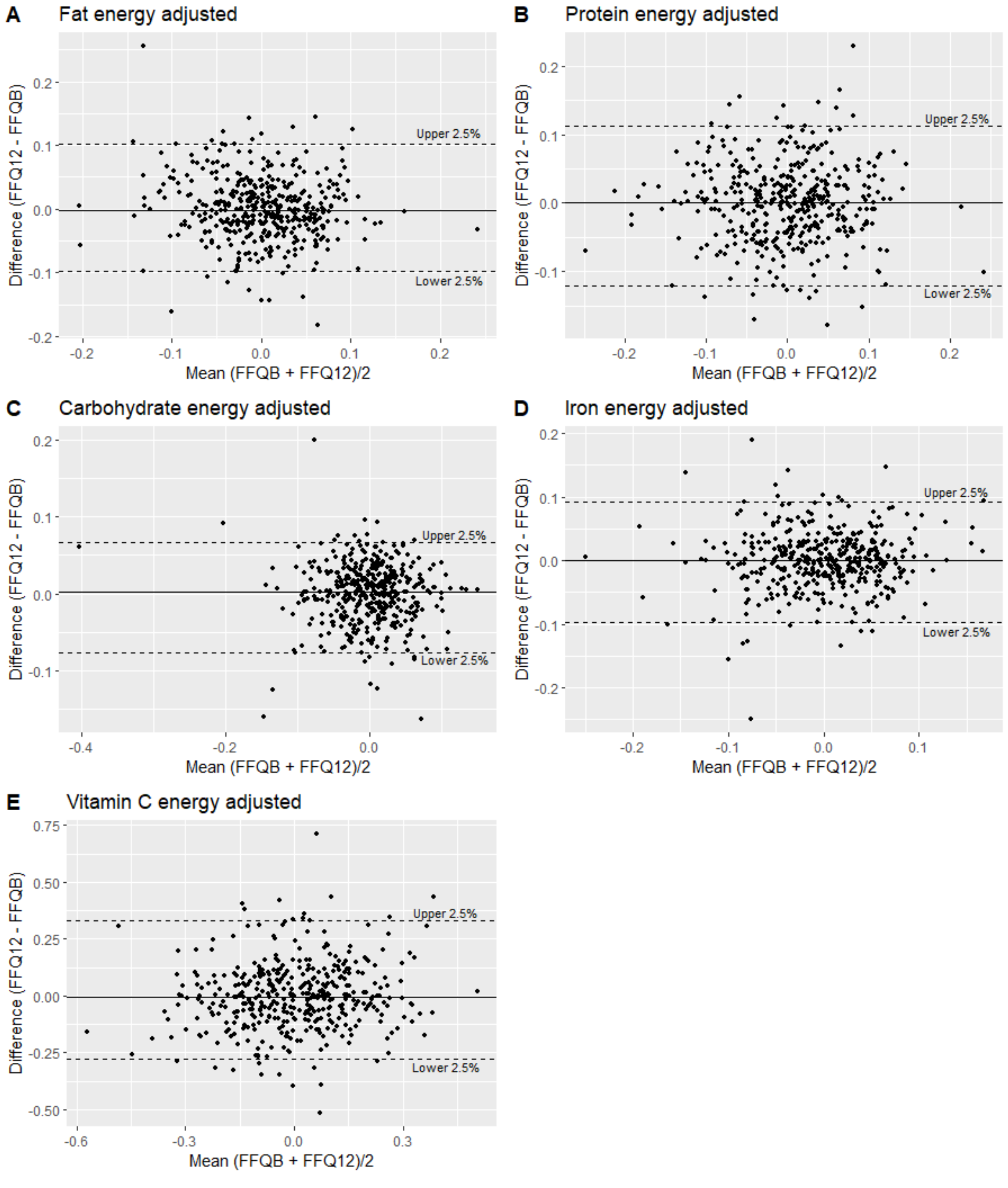

Figure S2. Bland-Altman plots comparing the $FFQ_{baseline}$ with the $FFQ_{12months}$ for nutrient residual intakes of: (**A**) fat, (**B**) protein, (**C**) carbohydrate, (**D**) iron and (**E**) vitamin C. The solid line illustrates the median difference, and the dotted lines illustrates the upper 2.5% and lower 2.5%. Energy-adjustment was performed using the residual method on double log-transformed nutrient intakes.
