# Supplementary material for: Relative Validity and Reproducibility of a Web-Based Semi-Quantitative Food Frequency Questionnaire in the Danish Diet, Cancer, and Health—Next Generations MAX Study
Source: Nutrients. 2023 May 19;15(10):2389. doi: 10.3390/nu15102389 (PMC10222214; doi:10.3390/nu15102389)
Supplement: Supplementary file 1 [file nutrients-15-02389-s001.zip › Table S1.pdf]

Table S1. Nutrient residual intakes from the $FFQ_{baseline}$ compared with the 24-HDRs for Wilcoxon sing-rank test, Bland-Altman (LOA), Spearman's correlation coefficient and cross-classification (n=289).

**Nutrients**[a]	**p-value**[c]	**Bland-Altman LOA**[d]		**Spearman's Correlation Coefficient**		**Cross-classification**			
		Lower	Upper	Crude (95% CI)	Energy-adjusted (95% CI)	Same quartile (%)	Adjacent quartile (%)	Opposite quartile (%)	Extreme opposite quartile (%)
Protein, g	0.9484	-29	42	0.26 (0.14-0.37)	0.48 (0.38-0.56)	41	38	17	4
Total fat, g	0.6999	-26	35	0.26 (0.14-0.38)	0.44 (0.34-0.52)	33	46	17	4
SFA, g	0.8726	-38	63	0.32 (0.21-0.42)	0.45 (0.35-0.54)	39	37	21	3
MUFA, g	0.7015	-38	63	0.23 (0.10-0.34)	0.37 (0.27-0.47)	36	38	20	6
PUFA, g	0.7704	-43	83	0.22 (0.11-0.33)	0.37 (0.26-0.47)	30	47	17	6
EPA, g[b]	0.2053	-[e]	-[e]	0.20 (0.09-0.31)	0.21 (0.10-0.31)	32	37	24	7
DHA, g[b]	0.1810	-[e]	-[e]	0.26 (0.15-0.37)	0.26 (0.15-0.37)	29	42	21	8
Cholesterol, mg	0.3378	-[e]	-[e]	0.31 (0.20-0.42)	0.37 (0.25-0.48)	35	39	19	6
Carbohydrate, g	0.4371	-23	37	0.35 (0.25-0.45)	0.49 (0.39-0.58)	40	37	19	4
Total sugar, g	0.4413	-[e]	-[e]	0.46 (0.35-0.55)	0.50 (0.39-0.59)	41	38	18	3
Fibre, g	0.8489	-44	82	0.43 (0.32-0.53)	0.53 (0.43-0.62)	41	40	16	3
Alcohol, g[b]	0.6808	-[e]	-[e]	0.55 (0.46-0.63)	0.46 (0.35-0.55)	47	33	15	5
Sodium, mg	0.9854	-[e]	-[e]	0.23 (0.11-0.33)	0.29 (0.18-0.39)	30	39	23	7
Potassium, mg	0.5604	-[e]	-[e]	0.29 (0.16-0.40)	0.42 (0.32-0.52)	35	41	20	4
Calcium, mg	0.9009	-47	86	0.28 (0.18-0.39)	0.38 (0.26-0.48)	35	43	16	6
Magnesium, mg	0.7779	-[e]	-[e]	0.36 (0.25-0.46)	0.55 (0.46-0.63)	40	42	15	3
Phosphorus, mg	0.5708	-34	55	0.28 (0.18-0.38)	0.37 (0.26-0.47)	38	36	20	6
Iron, mg	0.7368	-[e]	-[e]	0.30 (0.20-0.41)	0.40 (0.29-0.49)	35	41	18	6
Copper, mg	0.2163	-[e]	-[e]	0.51 (0.40-0.60)	0.56 (0.47-0.64)	43	43	12	3
Zink, mg	0.6166	-[e]	-[e]	0.23 (0.11-0.34)	0.29 (0.17-0.40)	33	39	21	7
Selenium, µg	0.8389	-[e]	-[e]	0.29 (0.17-0.39)	0.35 (0.23-0.44)	33	43	20	4
Iodine, µg	0.7304	-[e]	-[e]	0.30 (0.20-0.41)	0.28 (0.17-0.39)	39	31	22	8
Retinol, µg	0.9591	-[e]	-[e]	0.47 (0.36-0.56)	0.43 (0.33-0.53)	36	44	16	4
Beta carotene, µg	0.2237	-[e]	-[e]	0.39 (0.29-0.49)	0.37 (0.26-0.48)	36	43	13	7
Vitamin A, µg	0.5374	-[e]	-[e]	0.31 (0.19-0.41)	0.28 (0.16-0.39)	30	40	22	7
Vitamin D, µg	0.8621	-[e]	-[e]	0.25 (0.13-0.36)	0.28 (0.17-0.39)	35	35	23	8
Vitamin E, mg	0.4046	-[e]	-[e]	0.32 (0.21-0.42)	0.48 (0.39-0.58)	37	42	18	3

Vitamin K, µg[b]	0.0447	-[e]	-[e]	0.33 (0.22-0.42)	0.30 (0.20-0.40)	34	37	23	6
Thiamine, mg[b]	0.1096	-[e]	-[e]	0.28 (0.16-0.38)	0.29 (0.18-0.40)	37	38	17	7
Riboflavin, mg	0.5959	-40	83	0.34 (0.24-0.44)	0.43 (0.33-0.52)	40	37	19	3
Niacin, mg	0.9978	-[e]	-[e]	0.37 (0.25-0.47)	0.41 (0.31-0.50)	35	45	15	5
Vitamin B6, mg[b]	<0.0001	-[e]	-[e]	0.24 (0.12-0.35)	0.18 (0.07-0.29)	30	39	21	9
Vitamin B12, µg	0.4815	-[e]	-[e]	0.35 (0.25-0.45)	0.35 (0.23-0.45)	35	40	17	8
Vitamin C, mg	0.4815	-[e]	-[e]	0.36 (0.25-0.46)	0.42 (0.31-0.51)	36	40	20	4
Folate, µg[b]	0.5245	-[e]	-[e]	0.32 (0.21-0.42)	0.39 (0.31-0.51)	34	41	21	4

[a]Based on double log-transformed energy adjusted nutrient intake by the residual method. [b]EPA, DHA, alcohol, vitamin K, thiamine, vitamin B6 and folate are based on crude nutrient intake and afterwards energy-adjusted by the residual method. [c]p-value , test of difference in intake between FFQbaseline and the mean of 3x24-HDRs by Wilcoxon signed-rank test. [d]Bland-Altman limits of agreement (LOA) are reported as percentage difference. [e]Bland-Altman limits of agreement (LOA) are not reported as LOA depend on the level of the nutrient.
