# Supplementary material for: Relative Validity and Reproducibility of a Web-Based Semi-Quantitative Food Frequency Questionnaire in the Danish Diet, Cancer, and Health—Next Generations MAX Study
Source: Nutrients. 2023 May 19;15(10):2389. doi: 10.3390/nu15102389 (PMC10222214; doi:10.3390/nu15102389)
Supplement: Supplementary file 1 [file nutrients-15-02389-s001.zip › Table S2.pdf]

Table S2. Nutrient residual intakes from the $FFQ_{baseline}$ and the $FFQ_{12months}$ for Bland-Altman limits of agreement (LOA), Spearman's correlation coefficient and cross-classification (n=415).

**Nutrients**[a]	**p-value**[c]	**Bland-Altman LOA**[d]		**Spearman's Correlation Coefficient**		**Cross-classification**			
		Lower	Upper	Crude (95% CI)	Energy-adjusted (95% CI)	Same quartile (%)	Adjacent quartile (%)	Opposite quartile (%)	Extreme opposite quartile (%)
Protein, g	0.9900	-23	32	0.62 (0.55-0.69)	0.65 (0.58-0.71)	48	38	12	2
Total fat, g	0.7695	-21	25	0.70 (0.64-0.75)	0.64 (0.57-0.70)	48	38	12	2
SFA, g	0.7316	-30	39	0.69 (0.63-0.74)	0.68 (0.61-0.73)	49	39	11	1
MUFA, g	0.9923	-25	34	0.69 (0.63-0.74)	0.61 (0.54-0.67)	44	41	13	2
PUFA, g	0.9548	-31	46	0.69 (0.63-0.75)	0.66 (0.60-0.72)	48	41	9	1
EPA, g[b]	0.1712	-[e]	-[e]	0.71 (0.64-0.76)	0.63 (0.56-0.70)	48	39	9	3
DHA, g[b]	0.4834	-[e]	-[e]	0.72 (0.66-0.78)	0.65 (0.58-0.72)	48	40	8	3
Cholesterol, mg	0.9953	-[e]	-[e]	0.68 (0.63-0.74)	0.63 (0.56-0.70)	46	40	11	2
Carbohydrate, g	0.4255	-14	19	0.65 (0.59-0.71)	0.69 (0.62-0.74)	50	37	11	2
Total sugar, g	0.6321	-29	51	0.68 (0.61-0.74)	0.68 (0.62-0.74)	51	38	9	2
Fibre, g	0.9633	-32	48	0.69 (0.63-0.74)	0.74 (0.68-0.79)	53	40	7	1
Alcohol, g[b]	0.0128	-[e]	-[e]	0.81 (0.76-0.85)	0.74 (0.69-0.79)	52	40	7	1
Sodium, mg	0.8545	-26	36	0.61 (0.54-0.67)	0.57 (0.49-0.65)	46	40	12	3
Potassium, mg	0.6415	-23	27	0.66 (0.59-0.72)	0.69 (0.62-0.75)	49	41	8	2
Calcium, mg	0.8905	-41	66	0.69 (0.62-0.74)	0.72 (0.66-0.77)	56	35	7	2
Magnesium, mg	0.9294	-18	23	0.67 (0.60-0.73)	0.79 (0.73-0.83)	56	36	7	1
Phosphorus, mg	0.8158	-22	34	0.64 (0.57-0.70)	0.69 (0.63-0.74)	51	38	9	1
Iron, mg	0.8702	-19	25	0.65 (0.58-0.71)	0.72 (0.66-0.76)	52	39	8	1
Copper, mg	0.7008	-48	95	0.69 (0.63-0.74)	0.70 (0.63-0.75)	53	40	5	2
Zink, mg	0.8276	-20	27	0.62 (0.55-0.69)	0.64 (0.58-0.70)	47	38	14	1
Selenium, µg	0.7069	-[e]	-[e]	0.68 (0.61-0.73)	0.64 (0.57-0.71)	49	40	9	2
Iodine, µg	0.8123	-44	62	0.63 (0.56-0.69)	0.62 (0.55-0.68)	51	35	12	2
Retinol, µg	0.9969	-[e]	-[e]	0.68 (0.62-0.74)	0.67 (0.61-0.73)	48	41	9	2
Beta carotene, µg	0.8989	-63	162	0.70 (0.63-0.75)	0.66 (0.59-0.72)	50	37	11	2
Vitamin A, µg	0.7489	-51	96	0.65 (0.58-0.70)	0.52 (0.45-0.60)	43	37	16	4

Vitamin D, μg	0.9200	-[e]	-[e]	0.69 (0.62-0.74)	0.67 (0.60-0.73)	52	37	10	2
Vitamin E, mg	0.8330	-35	54	0.74 (0.69-0.79)	0.74 (0.68-0.79)	54	37	7	1
Vitamin K, μg[b]	0.4585	-299[f]	267[f]	0.73 (0.67-0.78)	0.66 (0.60-0.72)	49	40	9	2
Thiamine, mg[b]	0.9343	-0.46[f]	0.45[f]	0.62 (0.55-0.68)	0.63 (0.56-0.69)	48	38	11	3
Riboflavin, mg	0.9966	-33	44	0.68 (0.62-0.74)	0.74 (0.68-0.78)	55	37	7	1
Niacin, mg	0.8886	-27	39	0.71 (0.65-0.76)	0.71 (0.65-0.77)	49	41	9	1
Vitamin B6, mg[b]	0.5236	-0.76[f]	0.67[f]	0.64 (0.57-0.70)	0.62 (0.54-0.69)	50	36	11	3
Vitamin B12, μg	0.9035	-[e]	-[e]	0.64 (0.56-0.70)	0.61 (0.54-0.67)	50	34	13	2
Vitamin C, mg	0.5670	-53	90	0.69 (0.63-0.75)	0.64 (0.57-0.71)	49	38	9	4
Folate, μg[b]	0.2102	-301[f]	234[f]	0.70 (0.63-0.76)	0.69 (0.62-0.75)	51	39	7	2

[a]Based on double log-transformed energy adjusted nutrient intake by the residual method. [b]EPA, DHA, alcohol, vitamin K, thiamine, vitamin B6 and folate are based on crude nutrient intake and afterwards energy-adjusted by the residual method. [c]p-value , test of difference in intake between FFQbaseline and FFQ12months by Wilcoxon signed-rank test. [d]Bland-Altman limits of agreement (LOA) are reported as percentage difference. [e]Bland-Altman limits of agreement (LOA) are not reported as LOA depend on the level of the nutrient. [f]Bland-Altman limits of agreement (LOA) are reported as unit difference.
